# Harm reduction via online platforms for people who use drugs in Russia: a qualitative analysis of web outreach work

**DOI:** 10.1186/s12954-020-00452-6

**Published:** 2020-12-09

**Authors:** Arsen Davitadze, Peter Meylakhs, Aleksey Lakhov, Elizabeth J. King

**Affiliations:** 1 Centre for Health Economics, Management and Policy, National Research University — Higher School of Economics, 194100, Kantemirovstaya st. 3a, St. Petersburg, Russia; 2 St. Petersburg Charitable Fund «Humanitarian Action», Kamennoostrovsky prospekt, 63, A, 8-N, St. Petersburg, Russia; 3 University of Michigan School of Public Health, 1415 Washington Heights, SPH 1, Room 3842, Ann Arbor, MI 48104 USA

**Keywords:** Harm reduction, Web outreach, People who use drugs (PWUD), Online platforms, Qualitative research, Russia

## Abstract

**Background:**

Harm reduction services to people who use drugs (PWUD) in Russia are insufficient in terms of quantity, government endorsement, and accessibility. The situation has recently deteriorated even further because of social distancing measures of the COVID-19 pandemic. Several organizations have started to provide some harm reduction services via online platforms by web outreach. However, little is known on how online outreach services are organized and implemented. Drawing on the example of St. Petersburg-based NGO “Humanitarian Action,” we explored web outreach work in Telegram instant messenger.

**Methods:**

Our data were comprised of 4 semi-structured interviews with the NGO staff and 301 cases of web outreach work with PWUD. We used thematic analysis to study the process of web outreach, harm reduction service provision, and needs of PWUD.

**Results:**

Three stages of the process of web outreach work were identified: clients initiating communication, NGO workers addressing clients’ needs, and NGO workers receiving clients’ feedback. Communication proceeded in group chat or direct messages. Challenges in addressing clients’ needs happened when clients turned for help after hours, sent recorded voice messages, sent unclear messages, and/or were unwilling to transition to telephone communication. All web outreach workers reported receiving only positive feedback on their work. The needs of PWUD were categorized into two major themes, depending on whether they can be addressed fully or partially online. In cases of online only provision of services, web outreach workers helped PWUD treat minor injection drug use complications, obtain verified harm reduction information and receive general psychological support. In instances of partial online services provision, PWUD were assisted in getting treatment of severe injection drug use complications, overdoses, and in accessing offline medical, psychological, social, legal and harm reduction services.

**Conclusions:**

Our research demonstrated that web outreach work is a convenient tool for delivering some harm reduction services to PWUD either partially or completely online and for recruiting new clients (including hard-to-reach PWUD that avoid attending brick-and-mortar facilities). Harm reduction organizations should consider incorporating online harm reduction services into their activities. However, further research is needed to explore relative advantages and disadvantages of online harm reduction services.

## Introduction

People who use drugs (PWUD) are one of the most stigmatized and marginalized populations in Russia [1, 2]. People who inject drugs (PWID) are particularly vulnerable to HIV infection, HCV infection and fatal overdoses (OD) [3–7]. Unfortunately, harm reduction programs that have been proven to be effective for combatting HIV, HCV, and OD among PWID [8, 9] are not officially endorsed by the Russian government, their number is limited and access to them is low [10]. Moreover, the number of needle and syringe exchange programs (NSPs) in Russia has been decreasing since 2010 [11], while opioid agonist therapy (OAT) remains illegal [10]. In addition, harm reduction services were reported to be unattractive to young PWID in Russia [12].

Thus, there is an urgent need for alternative ways of providing harm reduction services to improve their accessibility among a wider population of PWUD in Russia. This is especially true for harder-to-reach populations, such as young PWUD. One such way to increase accessibility is by integrating harm reduction services into online platforms.

Darknet-based drug marketplaces are frequently used by PWUD. “Empire Market” [13], “Hydra” (in Russia) [14] and other darknet markets operate similarly to regular online marketplaces: they give vendors and buyers a platform to conduct their online transactions on. However, darknet-based drug marketplaces are more difficult to access as they require special software which encrypts computer IP addresses (e.g., Tor Browser). Transactions are anonymized and performed with cryptocurrencies (e.g., Bitcoin); hence, darknet markets are also called ‘cryptomarkets’. In addition to making purchases on the cryptomarkets, users can also exchange information on the availability of particular drugs, experiences from using them, their effects and potential harms via integrated online forums [15–17].

The use of drug marketplaces and drug-related online forums to facilitate harm reduction has started to gain the attention of researchers. A number of studies have shown that such online platforms could bring new opportunities to provision of harm reduction services [18–20]. Social media platforms have also shown potential to bring greater access to harm reduction services among PWUD [21]. Harm reduction interventions via online platforms are often referred to as ‘web outreach’, ‘online outreach’ or ‘netreach’ work [22, 23].

Web outreach work implies that harm reduction workers contact PWUD through online platforms and provide them with harm reduction information and counseling upon individual requests of users or distribute harm reduction information publicly via online forums. Such work helps to encourage risk reduction behaviors among hard-to-reach populations of PWUD, who do not attend brick-and-mortar harm reduction facilities [22, 23]. Moreover, the COVID-19 pandemic has introduced social distancing measures and shortages of medicines and harm reduction supplies, which makes it more difficult to provide in-person harm reduction services [24, 25]. Web outreach helps organizations continue to provide harm reduction services during the pandemic.

This research explores how web outreach work is organized and implemented by the oldest harm reduction NGO in Russia—“Humanitarian Action”. The aims of the study are: (1) to describe the process of online harm reduction provision; (2) to identify needs expressed by PWUD in Russia in the process of online harm reduction services provision; (3) to identify services provided for PWUD by web outreach workers. To our knowledge, this is the first study that explores web outreach work in terms of what needs of PWUD it helps to address and what services it helps to provide.

## Methods

### Setting

“Humanitarian Action” (henceforth, the NGO) is a non-governmental charitable organization based in St. Petersburg, Russia [26] that was established in 1997 and has been providing low-threshold harm reduction services for PWUD in Russia for over 25 years. Their services include exchange and disposal of used needles and syringes; provision of a motivational package containing sterile syringes, alcohol wipes, water for injections, ointments, bandages for PWUD; express testing for HIV, HCV, HBV, syphilis; support and referral of clients to the AIDS center for HIV diagnosis confirmation and start of antiretroviral therapy (ART); naloxone provision for opioid drugs overdose treatment; case management of PWUD in accessing social, medical and legal services [27]. Services are provided at no cost to PWUD, who come to the NGO’s mobile units, which circulate the city according to schedules posted on the NGO’s website [28].

The NGO recently added web outreach work to its activities in an effort to increase the program’s accessibility and extend its reach to hard-to-reach PWUD, who do not attend mobile units. The NGO uses two online platforms for provision of harm reduction services: the darknet forum in “Hydra” (the largest drug cryptomarket in Russia) and Telegram instant messenger (IM), which is popular in Russia. “Hydra” was established in 2015 and was reported to have a rapidly growing user base of over 2.5 million people in 2019 [14]. Telegram had over 400 million monthly active users worldwide (30 million in Russia) as of June 2020. Telegram is widely considered to be one of the most secure messengers for its end-to-end encryption method of communication, which facilitates greater anonymity of its users [29, 30]. As both platforms are commonly used means of communication among PWUD in Russia, it is reasonable to assume that provision of online harm reduction services with the use of these platforms will be convenient for them.

In January 2019, the NGO started its online program on “Hydra’s” drug forum. The organization’s own research of the forum’s content showed that it lacked information on HIV, HCV, and services provided by harm reduction organizations in Russia. The NGO’s deputy director was interviewed about the organization’s harm reduction services. This interview was published on “Hydra’s” Telegram channel. (In addition to the IM chat function, Telegram offers the opportunity to setup a “channel,” which is a newsfeed that people can subscribe to). The interview gathered around 100,000 views and became a part of web outreach work resulting in increased use of the NGO’s offline harm reduction services. Following the published interview, the NGO launched three threads on the “Hydra” forum dedicated to HIV, HCV and harm reduction services available in St. Petersburg. In addition to providing detailed information on the three topics, these threads serve as darknet-based platforms where users can interact with the NGO’s staff. Although these threads did not become popular among the forum’s users (more time and human resources are likely needed to increase their popularity among PWUD), they served as additional recruitment venues for services provided via Telegram IM.

In March 2019, the NGO started its web outreach work in Telegram IM. There, outreach workers of the NGO have their own channel as a way to communicate with subscribers and provide general information to PWUD. The NGO launched two venues in Telegram, including an open-access channel and a restricted-access chat. The open-access channel is a newsfeed available to anyone interested in what the NGO does. Its participants can find drug-related news, the NGO’s announcements and general harm reduction services information. Participants can only read the NGO’s posts there, but they cannot leave their own messages in the channel. The restricted-access chat was aimed to be more interactive and tailored to the needs of PWUD. At the initial stage of the chat’s launch, access was distributed among PWUD when they attended the NGO’s mobile units and through drug sellers in Telegram. The NGO asked sellers to promote the NGO’s services among their customers. Currently, access to the chat is granted to PWUD when they attend the mobile units. However, PWUD themselves are also able to provide access to the chat to their peers. This has helped to increase the chat’s popularity among PWUD. The chat had 1,125 members as of August 19, 2020. The chat functions as an online platform for PWUD where they can request various services from the NGO’s web outreach workers.

Web outreach workers at the NGO have good communication and community management skills as well as are experienced in using IMs and darknet platforms to communicate with PWUD. Web outreach workers are active or former drug users themselves. Each web outreach worker is qualified to provide a range of services to their clients. Some of them also specialize in working with cases related to their own background (for example, a web outreach worker who knows more about opioid misuse is more likely to reply to an opioid user’s request than other web outreach workers).

After the NGO started their online harm reduction services, they experienced an 89% increase in the number of requests for services in comparison with the time when only offline harm reduction services were provided [31].

### Data collection and research design

Data collection included 4 semi-structured interviews and 8 work reports of web outreach workers. The interviews with the NGO’s deputy director and 3 web outreach workers were conducted in June, 2020. The interview guide for the deputy director included topics related to the establishment and development of web outreach work, the current state of web outreach in the organization, and the organization’s plans for the future of web outreach. The interview guide for web outreach workers included experience in their web outreach work, ways of contacting clients, difficulties in communicating with clients, clients’ feedback on provided services, and perceived differences between online and offline harm reduction provision. Interviews were conducted in Russian and online via Zoom or Telegram. They were recorded and lasted around 30 min each. All participants provided written informed consent.

The researchers received copies of the work reports of web outreach workers from the NGO’s deputy director. The reports included detailed information on 301 cases of web outreach work, including date of contact (from November 2019 to June 2020), description of client’s requests, and description of services provided. The NGO provided only anonymized reports with respect to identities of PWUD and web outreach workers. No personal information of either was given to researchers.

Interviews were transcribed verbatim for data analysis. Thematic analysis [32] of interviews and work reports was conducted. An inductive approach to coding was used: two researchers (AD and PM) read interview texts and reports line by line thoroughly and assigned codes to each paragraph of interview transcripts and each line of work reports. Afterwards, one researcher (AD) combined the codes that conveyed similar meanings to determine a smaller set of initial themes. Microsoft Excel was used to organize the coding. An inductive approach was chosen to properly address the novelty of the studied topic and to produce an analysis deliberately anchored in empirical data. Further discussion of the initial themes by the two researchers led to clarification and elaboration of these themes on consensus basis. Thematic analysis of interviews helped researchers to explore how the NGO implements the web outreach processes. Thematic analysis of work reports assisted in identifying the needs of PWUD and understanding the extent to which and how these needs were addressed by web outreach workers. The Ethical Committee of the St. Petersburg Association of Sociologists approved the study.

## Results

### Process of web outreach

Thematic analysis revealed that the process of the NGO’s web outreach includes three distinct stages: initiating communication, addressing the client’s needs, and receiving feedback from the client. Within each of the stages, several important issues came up, including behaviors among PWUD, actions by outreach workers, and challenges faced.

The first stage of web outreach is PWUD initiating communication with web outreach workers. Different scenarios for this stage were described by the informants, depending on how PWUD contacted the NGO via Telegram.

The most common scenario for a communication commencement is when a PWUD sends her request in the NGO’s Telegram chat. After seeing the request, a web outreach worker contacts the PWUD in the chat or sends a direct message to her. If the web outreach worker contacts the PWUD in the chat, both the request and the worker’s response are displayed to the other members of the chat. If the web outreach worker contacts the PWUD via direct message, only the request is displayed to the other chat members. In this case, the worker’s response is seen only by the worker and the PWUD who sent the request. Web outreach workers reported that they chose between the two options of contacting the PWUD depending on the request’s content. For cases where requests regarded common information, workers chose to reply in the chat as the information potentially could be of use to the other chat members. For cases where requests required a more personal and confidential approach, workers preferred to contact the PWUD directly.

Sometimes when a PWUD sends his request in the chat, other PWUD address his request and start communicating with one another. Under such circumstances, web outreach workers join and monitor the discussion to guarantee that the request of the PWUD was addressed properly. The deputy director of the NGO notes that such instances support the development of a safe space and a sense of community among members of the chat.

“[Communication between PWUD] is friendly and fun, but it is under my strict supervision. As a moderator, I supervise such discussions and whenever rudeness appears […] I intervene and explain that this is not a space for insulting each other […] we want this space to be of maximum safety for you [PWUD].” (Deputy director of the NGO).

Telegram messenger allows its users to anonymize themselves by not disclosing any personal information to other users.

“What Telegram is good for is that you can hide your phone number, use any made-up name or a nickname, and start messaging […] you won’t be identified. Then you can write there [in the NGO’s Telegram chat] anything you want […] and you will get a response.” (Deputy director of the NGO).

In addition, web outreach workers told us that sometimes they are contacted by PWUD in Telegram outside of the NGO’s chat, via direct messages. In such cases, members of the chat cannot view the client’s requests and web outreach workers’ responses. One informant noticed that they were contacted more frequently in direct messages if they had recently been active in the NGO’s Telegram chat and/or channel.

“Sometimes questions [from PWUD] appear in response to newly published content by me [in Telegram channel and/or chat]. […] I noticed that the more often I respond [to clients’ messages in the chat], the more messages I get.” (Web outreach worker #3).

Another web outreach worker noted that some PWUD, who contacted her in direct messages but were not members of the NGO’s chat, got her contact information from the worker’s previous clients. Those clients seemed to advertise the worker’s services among their peers, thus increasing web outreach popularity among PWUD.

“They [PWUD] often message me saying, ‘my friend told me that […] you can give me some advice or teach me about something.’” (Web outreach worker #1)

The second stage of web outreach is the actual process of addressing clients’ needs by the NGO’s workers. Either the web outreach workers provide services to the clients themselves, or if they are unable to do it, they provide clients with contact information of other workers in the NGO, who are capable of assisting PWUD.

Telegram was described as a convenient “first step” of communication between a PWUD and a web outreach worker. These initial contacts via Telegram can open the door to harm reduction services being provided, both through further online consultations or referral to offline services.

“I find Telegram convenient, but it is just a start […] Comparing it to mobile units […] there is a door, which you enter […] then a window, where you get everything you need and get tested […] Telegram is the ‘door’, it is this first step […] It is what contact with a person starts from.” (Web outreach worker #2).

Informants mentioned several challenges related to the second stage of web outreach work, each of them concerning specifics of the services online provision. One challenge is that PWUD send their requests to web outreach workers after hours when web outreach workers are unable to provide immediate help. In such cases, the clients are contacted the next day and informed about times when workers are not available to promptly respond.

“We all need some time for ourselves. Any web outreach worker, including me, is sleeping at 4 AM. What you do is just explain [that you were unavailable] and, of course, provide services according to the request.” (Web outreach worker #3).

Another challenge arises when clients record voice messages instead of sending text messages. In such instances, web outreach workers may not have an opportunity to listen to voice messages promptly, thus the provided services are unlikely to be as immediate as the clients expect. Web outreach workers described that under such circumstances they asked clients if they could send a text message instead. Depending on their reply, counseling was offered after their text message was received or when the worker had an opportunity to listen to the voice message.

“If I get a voice message, I message the client, asking if it’s possible for them to send me a text message instead. If not, they have to wait. When I exit the subway, I’ll listen to it. Or I am working with other clients currently, and when I’m done with them, I’ll listen to it.” (Web outreach worker #3).

Another challenge arose when web outreach workers were unable to understand the content of the clients’ messages. Such occurrences happened when clients did not formulate their requests clearly. In these cases, the workers either tried to clarify the request by asking additional questions or suggested discussing the request by phone. Web outreach workers noted that some clients trusted them enough to discuss their requests on the phone, while other clients refused to continue their communication with a phone call and agreed to only text-form communication, as giving their phone number would compromise their anonymity. One informant reported that some clients stopped messaging her completely after a phone call was proposed:

“[After I suggest calling the PWUD] some of them send me their phone number or they ask me to send mine. Some of them exit the chat, and don’t reply there anymore.” (Web outreach worker #3).

Several web outreach workers mentioned phone calls as the next step in communication between them and some of their clients. Depending on the requests of PWUD, phone calls were favored as a continuation of communication by workers because in such instances, they were able to gain a better understanding of their clients’ emotions and, consequently, provide their services more effectively.

“Sometimes it is hard because you want real-life communication to understand what a person feels […] When you talk to them in person, you see their reactions, it’s easier to monitor some moments […] [for example,] whether they are ready to open up […] In Telegram, I don’t see the person and it’s hard for me to do my job to the fullest extent if a person doesn’t call me.” (Web outreach worker #2).

The third stage of web outreach is receiving client’s feedback on provided services. Web outreach workers described this stage as being crucial to their work, as it helps them to verify whether the services that they provided were of use for their clients and whether the clients knew whom to contact if they required help in the future. One worker narrated that it was very important for her to end communication with clients by leaving her contact information:

“This is my ‘message’: I always leave my phone number no matter what the request was, so that, just in case, they will know that they can call me.” (Web outreach worker #2)

All web outreach workers reported receiving only positive feedback on their work.

### Needs and services

In this section of the paper, we explore the first two stages of the process of web outreach work in terms of needs of PWUD and services provided to them. We identified two major themes in regard to whether the needs can fully be met or that the needs can only partially be met via online platforms. We distinguished several sub-themes under each theme based on common requests of PWUD and matched the sub-themes with services provided by web outreach workers.

#### Theme 1: Needs for online only harm reduction services

The first major theme is composed of needs for harm reduction services, which can be provided to PWUD entirely online. The following sub-themes were included in this theme: minor injection drug use complications; information regarding harm reduction, HIV and HCV; information regarding at-home detoxification; information regarding the COVID-19 pandemic; general psychological support.

The most common sub-theme was treatment of minor injection complications. Injection complications were defined as “minor” if their treatment did not require the offline assistance of a doctor. Such complications included collapsed veins, blown veins, venous ulcers, varicose veins, and rashes. PWID described their injection complications in text and/or by sending a photograph of their injury. PWID who requested help in such instances were offered advice from web outreach workers on how PWID could treat and prevent their injuries. Medical professionals verified the advice before web outreach workers sent it to clients. In cases where web outreach workers felt that more skilled assistance was needed, they provided PWID with contact information of doctors who work at the NGO. The doctors then would provide medical advice via Telegram.

Another common sub-theme was requesting harm reduction, HIV and HCV-related information. Nearly a sixth of requests made by PWUD concerned general information about harm reduction services provided at the NGO’s mobile units and the schedule of the mobile units. PWUD, who were taking antiretroviral therapy (ART) or wished to start ART, also requested information on the relevant schedule of the AIDS Center where ART is provided. A few clients requested information on transmission routes of HIV and HCV infections. The clients were provided with verified information on the topics, as well as with the contact information of other NGO workers, whom they could connect with should they have more specific questions.

A less common, yet still important, sub-theme was requesting information about at-home detoxification. Only five clients conveyed an interest in this topic. Web outreach workers articulated to such clients the potential risks of at-home detox, provided them with general information about medically assisted detox and shared contact information of workers, whom the clients could message regarding such issues.

Since April 2020, a number of COVID-19 information-related needs emerged. Several clients requested information on how they could enroll in a detoxification center and/or a rehabilitation program under the conditions of the pandemic. As in the previous cases, web outreach workers provided their clients with relevant and verified information in accordance with their requests.

A final sub-theme was that PWUD requested general psychological support via the Telegram IM. PWUD contacted web outreach workers to receive general psychological support concerning a number of personal issues. Common examples of requests included having difficulties in combatting drug addiction; expressing anxiety about repercussions of quarantine measures taken during the COVID-19 pandemic (e.g., shortages of medications); experiencing difficulties in coping with a partner’s death; and feeling anxiety about being infected with HCV. As the clients did not request help from a certified psychologist, web outreach workers provided them with advice based on their personal experience and contact information of the NGO staff whom they could message if they required additional help.

#### Theme 2: Needs for online and offline harm reduction services

The second major theme is comprised of harm reduction services, which cannot be provided to PWUD fully online, as some of their aspects require a client’s offline presence. This theme is represented with the following sub-themes of needs: getting medical, psychological, social, and legal services; severe injection drug use complications; getting harm reduction services; and drug overdoses.

The most common sub-theme was getting medical, psychological, social, and legal services. In nearly a third of cases in the dataset, PWUD requested help from the NGO in getting the following offline services: personal assistance in getting ART at the AIDS center, delivery of ART to a client’s home, personal assistance in being hospitalized at clinics for detoxification and rehabilitation, receiving counseling from a certified psychologist, obtaining identity documents, and obtaining disability status. In such cases, web outreach workers matched PWUD with other NGO staff, who specialize in working with these specific requests. PWUD were invited to the NGO mobile unit where outreach workers initiated face-to-face communication and the client’s needs were thus met offline.

Injection drug use complications in this theme were categorized as “severe” because their treatment required offline visits to a doctor. Such complications included severe cases of blown veins and venous ulcers, as well as edemas and skin abscesses. In such instances, web outreach workers referred PWID to doctors at the NGO as the workers themselves were not qualified enough to provide necessary help to their clients. The doctors contacted the clients online and after consulting them invited the PWID to the NGO mobile unit to receive treatment offline or encouraged them to go to a clinic to get necessary treatment.

Another major sub-theme was PWUDs requesting help in getting specific harm reduction services: HIV testing, HCV testing, harm reduction motivational packages, and PrEP medication. Web outreach workers could not address such needs via online platforms, thus they provided PWUD with instructions regarding how they could obtain such services offline at the NGO or affiliated clinics (in the cases of PrEP medication).

Overdoses (OD) were the least common sub-theme; only four cases of OD were mentioned in the work reports. In each case, the PWUD asked for help in treating an OD. Web outreach workers provided PWUD with a link to the NGO’s “Overdose bot” in Telegram. This bot, a built-in Telegram application created by the NGO, serves as an automated service, which provides Telegram users with information on symptoms of OD, cardiopulmonary resuscitation (CPR) techniques, and medications to treat an OD. It also provides them with contact information of a doctor, whom the PWUD can contact in order to get help online. Therefore, instead of web outreach workers manually searching and sending information to PWUD, they share a link with the PWUD, who then find the necessary information themselves using the bot. In each instance, web outreach workers provided their clients with a link to the bot. In two out of the four cases, PWUD had to call an ambulance for OD treatment. Thus, in addition to sending the link to the bot, the web outreach workers continued their communication with the clients until an ambulance arrived.

## Discussion

Our findings describe the process of web outreach work implemented by the NGO “Humanitarian Action,” a low-threshold harm reduction organization in Russia. Web outreach work to PWUD has only recently been implemented in Russia, a place that continues to struggle with meeting the need for harm reduction services. We described the stages of the web outreach process, needs of PWUD, who request help via online platforms, and services that are provided to them by web outreach workers. Our research demonstrates that a number of harm reduction-related needs among PWUD can be met entirely through web outreach work, while some can only be partially met online. These findings are in line with the existing literature on online platforms bringing new opportunities to harm reduction services provision [18–20]. They also contribute to the growing amount of literature regarding the processes of web outreach work [22, 23] and bring new evidence on how various needs of PWUD are addressed by web outreach services.

We identified a three-stage process of web outreach work. The process illustrates the benefits that PWUD gain from online harm reduction services provision without face-to-face contact with web outreach workers. An absence of requirement for physical presence of PWUD at a harm reduction organization facilitates greater level of anonymity in comparison with offline harm reduction services provision. In addition, the use of text messages brings greater convenience to PWUD, who do not feel comfortable with discussing drug use-related issues in person. These factors indicate that web outreach work helps to encourage harm reduction behaviors among PWUD who, otherwise, might not seek or have access to brick-and-mortar harm reduction services.

Our analysis of the needs of PWUD and services provided to them demonstrates two major functions performed by web outreach workers: 1. They can provide certain services completely online, and 2. They navigate clients within the organization in order to match the needs of the PWUD with a person who can address them. Our research on web outreach work indicates an increasing level of efficiency that comes from online provision of harm reduction services. Instead of traveling to a harm reduction facility, PWUD can contact the organization via an online platform. Furthermore, harm reduction services provided entirely online gain particular relevance amidst the COVID-19 pandemic when offline harm reduction organizations experienced new challenges to providing in-person outreach services.

Our findings suggest that online harm reduction services provision can be improved in terms of accessibility and efficiency. A challenge for web outreach work, as described by informants, was the inability of workers to communicate with PWUD after hours. One possible solution is to automatize some processes with Telegram bots, as it was done with the cases of OD. Currently, web outreach workers manually send information to PWUD. If automatized, then PWUD themselves could use a bot to get necessary information at any time of the day. However, not all services can be automatized with a bot; therefore, it may be necessary to employ some workers, who could reply to clients’ requests after hours. This is especially important in emergency situations, such as OD. Another way to develop provision of online harm reduction services is to increase their presence on darknet forums. Greater presence could potentially make online services accessible to more groups of PWUD, who request urgent help after hours and/or who do not use Telegram. Another obstacle in increasing accessibility of online harm reduction services was that some clients refused to continue communication with web outreach workers via the phone. More research is needed to explore the needs that PWUD have in such cases, identify the reasons why certain PWUD refuse to communicate via the phone, and explore how web outreach work can be provided in such instances.

Our research has several limitations worth noting. First, as anonymity of their clients is a priority for the NGO, all communications in direct messages are deleted a short time after work with them is completed. In some instances, workers filled their work reports after direct messages were deleted, which meant that they had to fill the reports based on memory. This implies a potential recall problem in descriptions of needs and services. Second, given that informants were asked to review their month-long work experience, another recall problem arises as a limitation of the study. For example, informants may have forgotten to mention more challenges of their web outreach work and cases of negative feedback. Third, web outreach work of the NGO in darknet forums was not included in the research due to its limited volume at the time of the study. Nonetheless, our findings provide rich descriptions of the novel web outreach work being done in Russia today. Our timely and descriptive findings can serve as the foundation and a reference point for further research into online harm reduction services as well as provide important information for existing organizations that seek to expand their harm reduction services to meet the needs of PWUD who may best be reached via online platforms.

## Conclusions

The findings from our research suggest that web outreach work is an approach that harm reduction organizations should consider implementing. Web outreach work may make provision of their services quicker, help to access hard-to-reach populations of PWUD and guarantee that services can be provided when offline provision cannot. However, web outreach work requires further research in order to explore its benefits for PWUD and harm reduction organizations. It is vital to examine which harm reduction services can be delivered entirely online rather than offline by estimating the net benefit for PWUD and harm reduction organizations. Such aspects as total anonymity and convenience of online platforms as well as potential loss in quality of services provision caused by online platforms should be considered. The positive net benefit would indicate that harm reduction organizations are to incorporate online harm reduction services provision into their activities or increase them. The web outreach work may be an important approach to help address challenges in reaching the younger generations of PWUD and also to ensure continuity of services during the COVID-19 pandemic and social distancing measures in place.

## Data Availability

The data used and/or analyzed during the current study is available from the corresponding author on reasonable request.

## Abbreviations

PWUD: People who use drugs
PWID: People who inject drugs
HIV: Human immunodeficiency virus
HCV: Hepatitis C virus
OD: Overdose
NSPs: Needle and syringe exchange programs
OAT: Opioid agonist therapy
NGO: Non-governmental organization
AIDS: Acquired immune deficiency syndrome
HBV: Hepatitis B virus
IM: Instant messenger
PrEP: Pre-exposure prophylaxis
